# Novel sequential ChIP and simplified basic ChIP protocols for promoter co-occupancy and target gene identification in human embryonic stem cells

**DOI:** 10.1186/1472-6750-9-59

**Published:** 2009-06-29

**Authors:** Ricardo B Medeiros, Kate J Papenfuss, Brian Hoium, Kristen Coley, Joy Jadrich, Saik-Kia Goh, Anuratha Elayaperumal, Julio E Herrera, Ernesto Resnik, Hsiao-Tzu Ni

**Affiliations:** 1 Dept. Antibody Applications and Stem Cells, R&D Systems, Inc., Minneapolis-MN, USA; 2 Dept. Molecular Biology, R&D Systems, Inc., Minneapolis-MN, USA

## Abstract

**Background:**

The investigation of molecular mechanisms underlying transcriptional regulation, particularly in embryonic stem cells, has received increasing attention and involves the systematic identification of target genes and the analysis of promoter co-occupancy. High-throughput approaches based on chromatin immunoprecipitation (ChIP) have been widely used for this purpose. However, these approaches remain time-consuming, expensive, labor-intensive, involve multiple steps, and require complex statistical analysis. Advances in this field will greatly benefit from the development and use of simple, fast, sensitive and straightforward ChIP assay and analysis methodologies.

**Results:**

We initially developed a simplified, basic ChIP protocol that combines simplicity, speed and sensitivity. ChIP analysis by real-time PCR was compared to analysis by densitometry with the ImageJ software. This protocol allowed the rapid identification of known target genes for SOX2, NANOG, OCT3/4, SOX17, KLF4, RUNX2, OLIG2, SMAD2/3, BMI-1, and c-MYC in a human embryonic stem cell line. We then developed a novel Sequential ChIP protocol to investigate *in vivo *promoter co-occupancy, which is basically characterized by the absence of antibody-antigen disruption during the assay. It combines centrifugation of agarose beads and magnetic separation. Using this Sequential ChIP protocol we found that c-MYC associates with the SOX2/NANOG/OCT3/4 complex and identified a novel RUNX2/BMI-1/SMAD2/3 complex in BG01V cells. These two TF complexes associate with two distinct sets of target genes. The RUNX2/BMI-1/SMAD2/3 complex is associated predominantly with genes not expressed in undifferentiated BG01V cells, consistent with the reported role of those TFs as transcriptional repressors.

**Conclusion:**

These simplified basic ChIP and novel Sequential ChIP protocols were successfully tested with a variety of antibodies with human embryonic stem cells, generated a number of novel observations for future studies and might be useful for high-throughput ChIP-based assays.

## Background

Regulatory transcription factors (TFs) are encoded by approximately 10% of the human genome [1]. The search for an accurate and complete list of target genes for thousands of TFs and the elucidation of their complex interactions at promoter sites, particularly in embryonic stem (ES) cells, has gained increasing interest. However, only a small fraction of the *in vivo *target genes and relatively few TF-TF interactions have been elucidated [2-4]. Chromatin immunoprecipitation (ChIP) and its derivatives (ChIP-chip, ChIP-seq, ChIP-SAGE, ChIP-PET, Sequential ChIP, etc) have been widely used for the investigation of TF-DNA interactions [4-9]. High-throughput approaches, such as ChIP-chip and ChIP-SAGE, are necessary for genome-wide analysis and the systematic identification of new DNA-binding sequences. Real-time (rt) PCR remains extensively used for validation of genome-wide data and for analysis of ChIP results in general. High-throughput approaches are time-consuming, expensive, labor-intensive, involve multiple steps that facilitate error introduction, and require complex statistical analysis [7,10]. Therefore, advances in this field will greatly benefit from the development and use of faster and straightforward ChIP assay and analysis methodologies.

Here, we present data obtained with a simplified, basic ChIP assay and analysis protocol that allowed the rapid identification of known target genes for SOX2, NANOG, OCT3/4, SOX17, KLF4, RUNX2, OLIG2, SMAD2/3, BMI-1, and c-MYC in the human ES cell line BG01V. We used rtPCR to initially validate the protocol/antibodies and densitometric analysis of PCR results with the ImageJ software as a more practical, less expansive, less time-consuming readout alternative. In addition, we developed a novel, non-disruptive, highly sensitive Sequential ChIP protocol for the identification of promoter co-occupancy, based on our simplified basic ChIP protocol. The data obtained with this Sequential ChIP protocol are consistent with data previously obtained with more labor-intensive, expensive, time-consuming ChIP-chip platforms. Furthermore, Sequential ChIP analysis led to the identification of two TF complexes in BG01V ES cells: SOX2/NANOG/OCT3/4/c-MYC and RUNX2/BMI-1/SMAD2/3 complexes. These two TF complexes associate with two different sets of target genes. The RUNX2/BMI-1/SMAD2/3 complex is associated predominantly with genes not expressed in undifferentiated BG01V cells, consistent with the reported role of those TFs as transcriptional repressors. These simplified basic ChIP and novel Sequential ChIP protocols were successfully tested with a variety of antibodies with BG01V ES cells, generated a number of novel observations for future studies and might be useful for high-throughput ChIP-based assays.

## Results

### Development of an improved basic ChIP protocol

**Figure 1:**
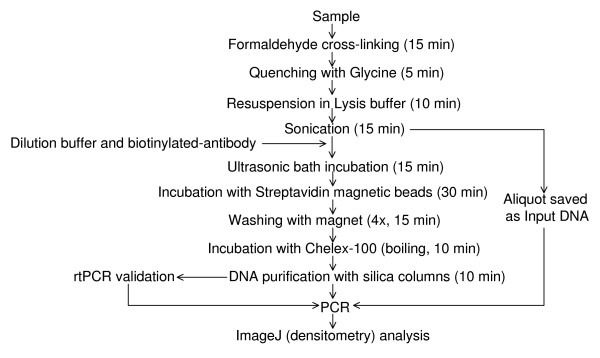
**Simplified, basic ChIP protocol schematic representation**.

We developed a simplified, basic ChIP protocol (diagram in Fig. 1) and test its usefulness with antibodies against TFs expressed in the human ES cell line BG01V. These antibodies included those against SOX2, NANOG, OCT3/4, SOX17, RUNX2, OLIG2, SMAD2/3, KLF4, BMI-1, and c-MYC. This basic ChIP assay is characterized by the combination of simplicity (several steps from conventional ChIP protocols were eliminated), speed (ChIP assay performed in about 2 hours; Fig. 1) and sensitivity (target genes easily detected with 20,000 cells or less). Recently described, commonly used protocols [11,12] normally take longer time or lack one or more of those characteristics. ChIP assays were performed with previously characterized antibodies [13] and known target genes and initially analyzed by rtPCR. We also analyzed the PCR results by densitometry using the ImageJ software, reducing time and resources for defining PCR parameters and, therefore, significantly decreasing experimental costs. Target genes included *FGF4*, *LEFTY*, *NANOG*, *VEGF*, *BCL2*, *GLI1*, *E-CADHERIN*, *OCT3/4*, *c-MYC*, *HESX1*, *ZFP206*, and *SUZ12 *(SOX2/NANOG/OCT3/4 targets), *LAMA1 *(SOX17), *B2R *(KLF4), *HOXC13 *(BMI-1), *c-MYC *and *GLI1 *(SMAD2/3), *P21 *(OLIG2) and *VEGF *and *BAX *(RUNX2). All primer sets have been validated previously (Table 1) [14-37]. Normal IgG and input DNA (0.1% of whole cell lysate) were used as negative controls.

**Table 1:** Primer sets used in PCR/rtPCR reactions.

Promoter	Primer sequences (Forward/Reverse)	Reference
*NANOG*	GTCTTTAGATCAGAGGATGCCCC/CTACCCACCCCCTATTCTCCCA	[14]
*c-MYC*	GAAGCCTGAGCAGGCGGGGCAGG/GCTTTGATCAAGAGTCCCAG	[15]
*BCL-X*	CTGCACCTGCCTGCCTTTGC/GGAGAGAAAGAGATTCAGGA	[16]
*P21*	CCAGCCCTTGGATGGTTT/GCCTCCTTTCTGTGCCTGA	[17]
*SUZ12*	TCACCCTACCCTGGCCTCGCT/TCGCTAAACCGCTCGCTGGGT	[18]
*MUC4*	AAACTAGGGACTCCTACTTG/GGACAGAATGGGGTGAAT'	[19]
*FOS*	GGCGAGCTGTTCCCGTCAATCC/GCGGGCGCTCTGTCGTCAACTCTA	[20]
*HOXC13*	TGCAGCGGAGCGAGCCCC/TCAACAGGGATGAGCGCGTCGTG	[21]
*GLI1*	CTCGCGGGTGGTCCGGGCTTG/CCGCCTGCCCCCCCTTCTCA	[22]
*BCL2*	CAGTGGGTGGCGCGGGCGGCA/CCCGGGAGCCCCCACCCCGT	[23]
*E-CADHERIN (CDH1)*	TAGAGGGTCACCGCGTCTAT/TCACAGGTGCTTTGCAGTTC	[24]
*OCT3/4*	TGAACTGTGGTGGAGAGTGC/AGGAAGGGCTAGGACGAGAG	[14]
*FGF4*	GGGAGGCTACAGACAGCAAG/CTGTGAGCCACCAGACAGAA	[14]
*LEFTY*	AAGCTGCAGACTTCATTCCA/CGGGGGATAGATGAAGAAAC	[14]
*VEGF*	CCTCAGTTCCCTGGCAACATCTG/GAAGAATTTGGCACCAAGTTTGT	[25]
*SNAIL*	GGCGCACCTGCTCGGGGAGTG/GCCGATTGCCGCAGCA	[26]
*PTEN*	CCGTGCATTTCCCTCTACAC/GAGGCGAGGATAACGAGCTA	[27]
*SMA*	AGCCAAGCACTGTCAGGA/ACAATGGATGGGAAAACAG	[28]
*COL2A*	TTCCAGATGGGGCTGAAAC/ATTGTGGGAGAGGGGGTCT	[29]
*GATA4*	ACAGGAGATGGGAAGTGTCGC/GGTGACCTCTTGGGCTCAACTC	[30]
*GATA6*	CATTTCCAGTCCCTTTTGCCC/TTCCACATCAGTCGTGTCCGAG	[30]
*BAX*	ACAGTGGCTCACGCCTGTAAT/AGCCTCCCAAGTAGCTGGAATTG	[31]
*TGFB*	GTGCAGCAAAAGAGGCTGCGTGCG/TCTATTTCTCTCTGCTGAAAT	[32]
*B2R*	GCAGAGCGGAGAGCGAAGG/GCCTGATGTCCCCACCGTC	[33]
*IL2*	CGTTAAACAGTACCTCAAGCTCAA/CCTTTTTATCCACACAAAGAGCTA	[34]
*ZFP206*	CCGGCCAGATTTCACTAAAGAGC/CCTACCCCATGAAATTTTGCCAG	[35]
*GAPDH*	GTGTTCCTACCCCCAATGTGT/ATTGTCATACCAGGAAATGAGCTT	[14]
*HESX1*	GTGTTCATTGACATGCTAA/GGACCAGAAGAAAGACTGTG	[36]
mouse Lama1*	CCTCAGCTCCAAGAAAGGAG/AGGATGCTTCCCTGAAATCC	[37]

* All others are human genes.

**Figure 2:**
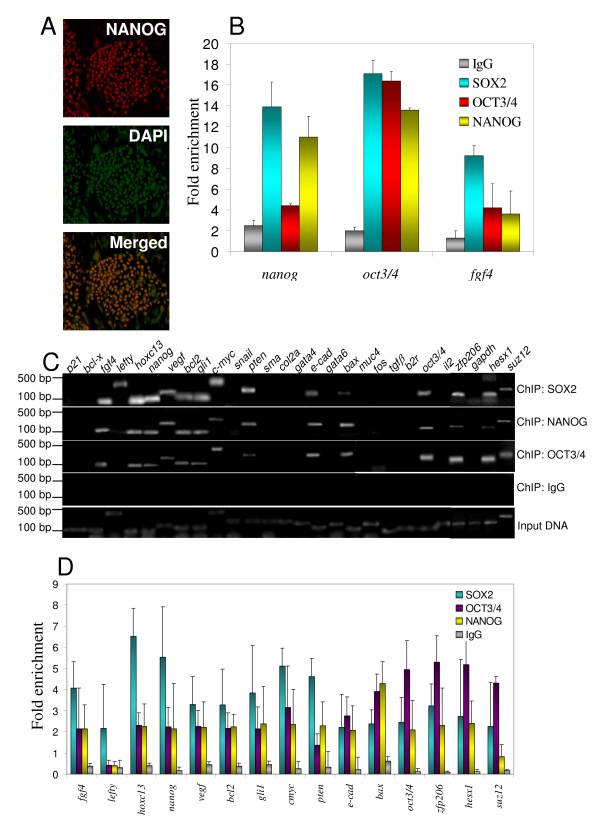
**Identification of SOX2/NANOG/OCT3/4 target genes using the simplified, basic ChIP protocol**. (A) NANOG expression by immunocytochemistry (red), DAPI (green) and merged images of undifferentiated BG01V human embryonic stem cells (magnification = 20×). (B) rtPCR data from SOX2/NANOG/OCT3/4 ChIP using primers to previously known target genes. (C) PCR results from the indicated ChIP assays with undifferentiated BG01V cells. (D) Densitometric analysis of ChIP data shown in C, obtained with ImageJ software. Three independent experiments were performed. Representative results are shown. Fold enrichment = ChIP/Input DNA. Error bars represent standard deviation.

The undifferentiated human ES cell line BG01V, used throughout this study, shows NANOG expression and nuclear localization (Fig. 2A, merged image), as expected [38]. SOX2 and OCT3/4 expression and nuclear localization was also verified (data not shown). Fig. 2B shows fold enrichment relative to input DNA, as determined by rtPCR analysis, of three known SOX2/OCT3/4/NANOG target genes (*OCT3/4*, *NANOG*, and *FGF4*) [10,39] from SOX2/OCT3/4/NANOG ChIP assays. Fig. 2C depicts PCR results using 28 candidate targets and Fig. 2D shows fold enrichment, as determined by densitometric analysis, of the PCR results (Fig. 2C), of the three abovementioned known targets (*OCT3/4*, *NANOG*, and *FGF4*) and additional known SOX2/OCT3/4/NANOG targets (*LEFTY*, *VEGF*, *BCL2*, *GLI1*, *E-CADHERIN*, *c-MYC*, *HESX1*, *ZFP206*, and *SUZ12*) [10,39]. Figs. 2C and 2D also suggest *HOXC13*, *PTEN *and *BAX *as novel putative SOX2/OCT3/4/NANOG targets in undifferentiated BG01V cells (only positively detected promoters are shown in the graph). These results indicate that the described ChIP assay and analysis protocol are suitable for ChIP screening. Moreover, the results also suggest that the densitometric quantification of PCR results using the ImageJ software can be used as an alternative ChIP readout, reducing experimental time and resources.

**Figure 3:**
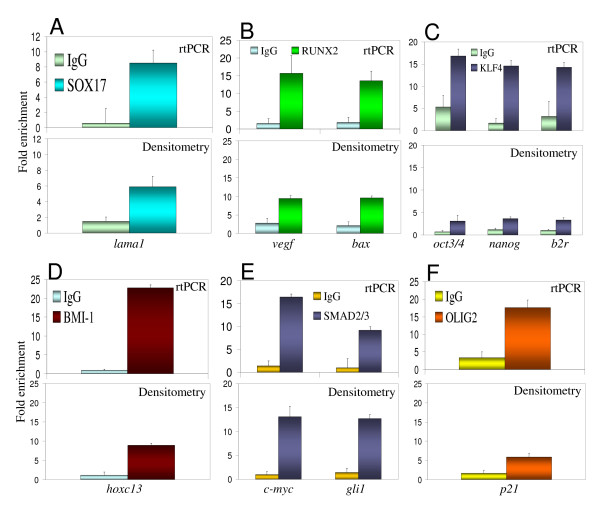
**Identification of known target genes for a variety of stem cell transcription factors using the simplified, basic ChIP protocol**. (A) Mouse *Lama1 *detection from SOX17 ChIP using endoderm differentiated D3 mouse embryonic stem cells. (B) *VEGF *and *BAX *detection from RUNX2 ChIP. (C) *OCT3/4*, *NANOG*, and *B2R *detection from KLF4 ChIP. (D) *HOXC13 *detection from BMI-1 ChIP. (E) *c-MYC *and *GLI1 *detection from SMAD2/3 ChIP. (F) *P21 *detection from OLIG2 ChIP. (A) - (F): Fold enrichment = ChIP/Input DNA. Error bars represent standard deviation. (B-F): using undifferentiated BG01V human embryonic stem cells.

To further confirm the usefulness of the protocol and test its applicability, ChIP assays were performed with additional ES cell TFs and their known target genes. Expression and nuclear localization of all these TFs were verified (data not shown). SOX17 plays critical roles in development regulation, stem cell function and is required for endoderm specification and maintenance in the embryo [40]. As expected [41] the mouse *Lama1 *promoter was detected by rtPCR and densitometric analysis (Fig. 3A) from SOX17 ChIP assays using endoderm differentiated mouse D3 ES cells. RUNX2, linked to bone development and maintenance [42], regulates *VEGF *and *BAX *[31,43], which were detected by rtPCR and densitometric analysis (Fig. 3B) from RUNX2 ChIP assays using undifferentiated BG01V cells. KLF4, implicated in transcriptional repression in smooth muscle cells, kidney development, stem cell self-renewal and pluripotency [33,44,45], regulates *OCT3/4*, *NANOG*, and *B2R *[33,45], which were detected by rtPCR and densitometric analysis (Fig. 3C) from KLF4 ChIP assays using undifferentiated BG01V cells.

BMI-1, implicated in cell proliferation and stem cell self-renewal [46,47], binds to the *HOXC13 *promoter [21], which was detected by rtPCR and densitometric analysis (Fig. 3D) of BMI-1 ChIP assays using undifferentiated BG01V cells. SMAD2/3, involved in epithelial-to-mesenchymal transition, neuronal differentiation, and chondrogenesis [29,48-50], have been shown to regulate *c-MYC *and *GLI1 *[50], which were also detected by rtPCR and densitometric analysis of SMAD2/3 ChIP assays using undifferentiated BG01V cells (Fig. 3E). OLIG2, implicated in neuron, astrocyte and oligodendrocyte differentiation, binds to the *P21 *promoter [51], which was detected by rtPCR and densitometric analysis (Fig. 3F) of OLIG2 ChIP assays using undifferentiated BG01V cells.

**Figure 4:**
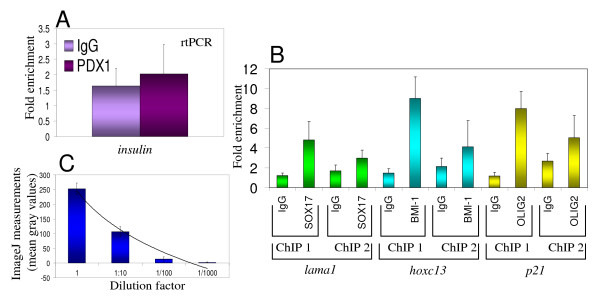
**Specificity and applicability of the simplified, basic ChIP protocol**. (A) As an additional negative control, the insulin promoter could not be detected from PDX1 ChIP assays using undifferentiated BG01V human embryonic stem cells. (B) The simplified, basic ChIP protocol (ChIP 1) was directly compared to previously described [20] commonly used ChIP protocol (ChIP 2), using three different antibodies (SOX17, BMI-1, OLIG2) and their respective known target genes (*Lama1*, *HOXC13*, *p21*). (C) ImageJ measurements (mean gray values) of PCR products from OLIG2 ChIP assays (*p21 *promoter detection), using a serial dilutions of DNA sample (from OLIG2 ChIP) and *p21 *promoter primers (logarithmic curve was added to the bar graphic). Fold enrichment = ChIP/Input DNA. Error bars represent standard deviation.

As an additional negative control, an antibody against PDX1, which is involved in pancreatic organogenesis [52], was also used in ChIP assays. As expected [52] PDX1 was not detected by immunofluorescence in undifferentiated BG01V cells (data not shown). Consistently, a know PDX1 target gene, insulin [52], could not be detected above background by rtPCR in PDX1 ChIP assays performed with undifferentiated BG01V cells (Fig. 4A). In addition, our basic ChIP protocol was directly compared to a previously described [20] commonly used ChIP protocol, using three of the above mentioned antibodies and their target genes. Fig. 4B shows that our basic ChIP protocol leads to higher fold enrichment, compared to IgG controls, than the fold enrichment obtained with a previously described [20] commonly used ChIP protocol. Finally, to add experimental evidence for the applicability of the densitometric analysis of ChIP results using the ImageJ software, OLIG2 ChIP assays were performed with serially diluted template and primers. Fig. 4C shows that ImageJ measurements of *P21 *promoter signals from these OLIG2 ChIP assays are within a logarithmic range.

Taken together, these results confirm that our simplified, basic ChIP assay can be used with a variety of antibodies and that the densitometric quantification of PCR results using the ImageJ software can be used as an alternative ChIP readout.

### SOX2/OCT3/4/NANOG/c-MYC complex co-occupies several promoters in BG01V cells

**Figure 5:**
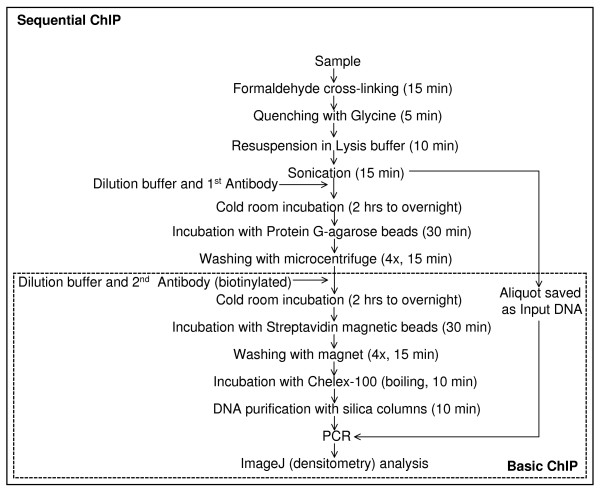
**Sequential ChIP assay schematic representation**.

We then sought to examine TF-TF interactions at promoter sites (promoter co-occupancy) using a newly developed Sequential ChIP assay (diagram in Fig. 5), which is based on the simplified basic ChIP assay. ChIP-chip studies have identified numerous promoters co-occupied by the SOX2/OCT3/4/NANOG complex [10,39]. To validate the effectiveness of the Sequential ChIP assay we selected a number of SOX2/OCT3/4/NANOG known targets and subjected these targets to our new protocol.

**Figure 6:**
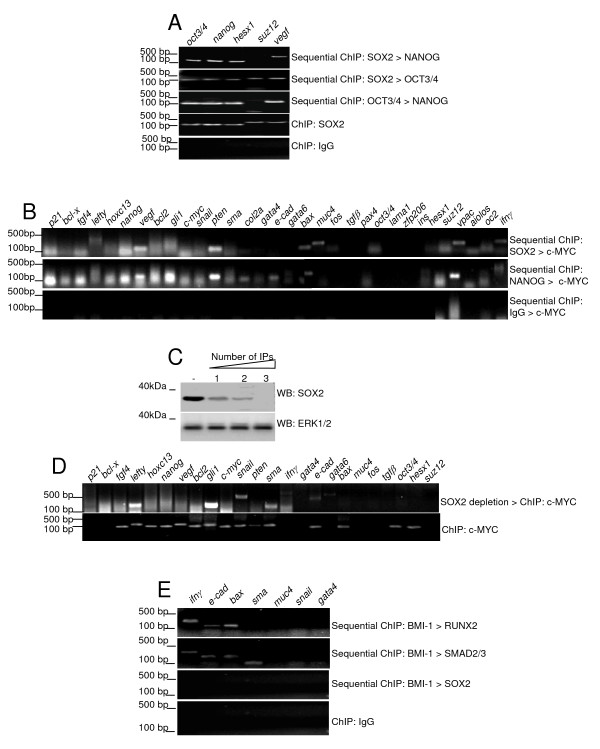
**Identification of SOX2/OCT3/4/NANOG/c-MYC and RUNX2/BMI-1/SMAD2/3 as complexes in BG01V cells using the novel Sequential ChIP protocol**. (A) Detection of promoter co-occupancy by SOX2/OCT3/4/NANOG, with the indicated combinations of antibodies. (B) Detection of promoter co-occupancy by SOX2/OCT3/4/NANOG/c-MYC, with the indicated combinations of antibodies. (C) SOX2 immunodepletion of BG01V lysates by three subsequent rounds of SOX2 immumoprecipitation, using ERK1/2 as a negative control. (D) c-MYC ChIP assays results using SOX2-depleted or non-depleted BG01V human embryonic stem cell lysates. (E) Detection of promoter co-occupancy by BMI-1/RUNX2/SMAD2/3, with the indicated combinations of antibodies.

The data obtained with the Sequential ChIP assay in undifferentiated BG01V cells is consistent with previously reported ChIP-chip data [10,39]. First, these three TFs (SOX2/OCT3/4/NANOG) have been reported to co-occupy the *OCT3/4*, *NANOG*, *HESX1 *and *VEGF *promoters [10,39], and consistent with that, any combination of those TFs antibodies in the Sequential ChIP assay led to the detection of those promoters (Fig. 6A). Second, only SOX2 and OCT3/4 co-occupy the *SUZ12 *promoter [10], and consistent with that, only immunoprecipitation with the SOX2 antibody followed by the OCT3/4 antibody lead to the detection of the *SUZ12 *promoter (Fig. 6A, middle panel, "Sequential ChIP: SOX2 > OCT3/4"), whereas other antibody combinations did not. A SOX2 regular ChIP assay was used as a positive control (Fig. 6A).

SOX2/OCT3/4NANOG and c-MYC have been found to share a large number of target genes in ChIP-chip studies [10,39,53-55]. We then investigated if c-MYC co-occupies promoters with the SOX2/OCT3/4/NANOG complex. Indeed, immunoprecipitation with the SOX2 antibody followed by the c-MYC antibody led to the detection of a number of promoters in undifferentiated BG01V cells (Fig. 6B, top panel, "Sequential ChIP: SOX2 > c-MYC"). The same promoters were detected if the NANOG antibody preceded the c-MYC antibody (Fig. 6B, middle panel, "Sequential ChIP: NANOG > c-MYC") or if the OCT3/4 antibody preceded the c-MYC antibody (data not shown). In contrast, none of these promoters were detected when normal IgG was used as the first antibody (Fig. 6B, last panel).

These results indicate that, at the indicated promoters (Fig. 6B), c-MYC is a subunit of the SOX2/OCT3/4/NANOG complex in undifferentiated BG01V cells. To further test this hypothesis we immunodepleted SOX2 from undifferentiated BG01V cells lysates (Fig. 6C, ERK1/2 as negative control) and then analyzed c-MYC targets by ChIP. Fig. 6D shows which c-MYC-bound promoters were detected in those SOX2-depleted lysates and shows a significant reduction in the number of c-MYC-associated promoters. Most of the promoters detected in Fig. 6B top panel ("Sequential ChIP: SOX2 > c-MYC") were not detected by c-MYC ChIP after SOX2 depletion (Fig. 6D), including *P21*, *FGF4*, *NANOG*, *VEGF*, *PTEN*, *BAX*, *MUC4*, *OCT3/4 *and *SUZ12 *(densitometric analysis confirmed that these signals were above input DNA levels in Fig. 6B but not in Fig. 6D, data not shown).

Collectively these results corroborate the applicability of the novel Sequential ChIP assay. In addition these results indicate that c-MYC associates with the SOX2/OCT3/4/NANOG complex in BG01V cells.

### RUNX2/BMI-1/SMAD2/3 complex co-occupies a second set of promoters in BG01V cells

We then sought to investigate the presence of other TF complexes in BG01V cells using the Sequential ChIP assay. In a screening with 25 potential target genes, we found that BMI-1, RUNX2, SMAD2/3, KLF4 and OLIG2 bind to a similar set of promoters in BG01V cells (data not shown). Therefore, we use this group of TFs in Sequential ChIP assays. Seven of the promoters that were detected by most or none of those TFs were selected for this set of experiments. Our results indicate that *IFNG*, *E-CADHERIN*, and *BAX *promoters are co-occupied by a BMI-1/RUNX2/SMAD2/3 complex, and that the *SMA *promoter is co-occupied by BMI-1/SMAD2/3 (Fig. 6E). The *GATA4 *promoter was not detected in any of the individual ChIP assays for this group of TFs (data not shown) and was used as an additional negative control. Consistently *GATA4 *was not detected by Sequential ChIP using any combination of antibodies (Fig. 6E). In addition, *MUC4 *was not detected when BMI-1 was the first antibody in the Sequential ChIP assay (Fig. 6E), as expected, since *MUC4 *is not detected in BMI-1 ChIP assays (data not shown). Furthermore, Fig. 6E shows that *SNAIL *was not detected when the BMI-1 antibody was followed by RUNX2 or SMAD2/3 antibodies, which was also expected, since *SNAIL *was detected in BMI-1 ChIP assays, but not detected in RUNX2 or SMAD2/3 ChIP (data not shown). None of the above mentioned promoters were detected when the BMI-1 antibody was followed by the SOX2 antibody (Fig. 6E) or by the KLF4 or OLIG2 antibodies (data not shown). Finally, none of the above mentioned promoters were detected when the SMAD2/3 antibody was followed by the KLF4 or OLIG2 antibodies (data not shown).

Overall, these experiments suggest the presence of a RUNX2/BMI-1/SMAD2/3 complex at the above mentioned promoters in undifferentiated BG01V cells.

## Discussion

Results shown here indicate that our simplified, basic ChIP and the novel Sequential ChIP assays are suitable for systematic target gene identification and detection of TF interactions at specific DNA sites. The suitability of the basic ChIP assay was confirmed by our ability to rapidly identify known targets of SOX2/OCT3/4/NANOG (*FGF4*, *LEFTY*, *NANOG*, *VEGF*, *BCL2*, *GLI1*, *E-CADHERIN*, *OCT3/4*, *c-MYC*, *HESX1*, *ZFP206*, and *SUZ12*) [10,35,36,39], SOX17 (mouse *Lama1*) [41], RUNX2 (*VEGF *and *BAX*) [31,43], KLF4 (*OCT3/4*, *NANOG*, *B2R*) [33], BMI-1 (*HOXC13*) [21], SMAD2/3 (*VEGF*, *IFNG *and *FOS*) [50], and OLIG2 (*P21*) [51].

The basic ChIP protocol used here is a simplified, combined version of fast ChIP protocols previously described [11,12]. The main advantages of this basic ChIP assay over previously published conventional protocols [[20,31,37], for example] is the combination of speed (only about 2 hours to obtain ChIP samples, via the elimination of some common steps) and sensitivity (known targets could be detected with only about 20,000 cell equivalents were used per PCR reaction). Our basic ChIP protocol combines biotinylated antibodies, streptavidin magnetic beads, chelating resin for elimination of PCR inhibitors [11], and DNA purification with commercially available silica columns. It differs from previously described fast ChIP protocols [11,12] by not involving: antibody capture with protein A, cross-linking reversal, proteinase K treatment and phenol-chloroform-ethanol treatment for DNA isolation. In preliminary experiments we noticed that the combination of chelating resin [11] and silica columns for DNA purification leads to greater PCR products yields than with chelating resin alone (data not shown). The biotin-streptavidin interaction represents one of the strongest non-covalent interactions known [56] and therefore it represents an advantage over protein A-IgG interaction [11]. Our preliminary observations (data not shown) and those reported by other investigators [11,12] suggest that cross-linking reversal and proteinase K treatment can be avoided, although it might result in a slightly lower DNA yield. The boiling of the samples with chelating resin and subsequent DNA purification with silica columns seem to replace the need for cross-linking reversal and proteinase K treatment.

The use of densitometric analysis of PCR results qualitatively matched the rtPCR results for all the antibodies used here since all above mentioned known targets were detected above background by both densitometry and rtPCR (Figs. 2 and 3). Densitometric analysis with the ImageJ software, due to its simplicity, practicality and less time and resources used in preliminary calibration experiments, offers a viable alternative to rtPCR when absolute quantification is not required. In addition, it can be applied for pre-screenings prior to rtPCR. It was expected, however, that densitometric analysis with the ImageJ software would show a quantification limitation, probably coming from pixel saturation in DNA bands from PCR reactions and consequent lower measurement capacity. Such limitation probably caused the lower levels of fold enrichment observed in the densitometric analysis as compared to rtPCR for some of the ChIP assays (Fig. 3; KLF4, BMI-1 and OLIG2 ChIP assay). However, our comparisons indicate that when the goal is simply the identification of target genes, densitometric analysis provides a reliable alternative, besides, for some antibodies, such limitation was minimum (Fig. 3; SOX17 and SMAD2/3 ChIP assays).

We then analyzed promoter co-occupancy using the newly developed Sequential ChIP assay (diagram in Fig. 5). This assay provides a significant improvement from previously described Sequential ChIP assays [57-60] and it differs from those in two main aspects. First, during the Sequential ChIP assay the antibody-antigen interaction is never disrupted by epitope-specific peptides or other treatments, and consequently immunoprecipitations can be carried-out in the same, intact samples. This feature might contribute for lower background, higher specificity and the possibility of using smaller samples. Second, the modifications introduced in the basic ChIP assay to make it a straightforward, fast, sensitive assay, were maintained in the Sequential ChIP protocol. In addition, the Sequential ChIP combines two methods of physical separation of the targeted DNA fragments. The first antibody, non-biotinylated, binds to agarose beads, via protein G-IgG interaction, and is brought down by centrifugation. The second antibody, this time a biotinylated one, binds to magnetic beads, via biotin-streptavidin interaction, and is separated from the rest of the sample with a magnet. These two forms of physical separation (gravity versus magnetism) are quite complementary and exert minimum interference over each other. The results are even visual with the reagents used here: when the antibodies used in the assay recognize TFs that co-occupy a significant number of target genes, as it is the case for SOX2/OCT3/4/NANOG/c-MYC, one can see the agarose beads slurry attaching to the magnet in the last steps of the assay, since they are carried by the streptavidin-magnetic beads. This visual effect is not observed with any of the negative controls. In proof-of-principle experiments we investigated promoter co-occupancy in undifferentiated BG01V cells using SOX2, OCT3/4 and NANOG antibodies (Fig. 6A) and the obtained results are consistent with those obtained in ChIP-chip studies [10,39].

Furthermore, our Sequential ChIP assay identified c-MYC as a potential interacting partner of the SOX2/OCT3/4/NANOG complex at several gene promoters in undifferentiated BG01V cells (Fig. 6). It has been suggested that c-MYC is involved in the regulation of up to 15% of all genes. It has been reported that c-MYC modulates global chromatin structure via its interaction with histone acetyl-transferase (HAT) and SWI/SNF chromatin remodeling complexes [61-63], which might be c-MYC function in the SOX2/OCT3/4/NANOG complex. Supporting this hypothesis, SOX2/OCT3/4/NANOG and c-MYC have been found to share a large number of target genes in ChIP-chip studies [10,39,53-55].

Furthermore, data shown in Fig. 6E suggests the presence of a RUNX2/BMI-1/SMAD2/3 complex in undifferentiated BG01V cells. Interestingly, the association of SMAD2/3 with RUNX2 has been reported previously in B-lymphocytes and human breast cancer cells [64,65]. BMI-1 is part of the Polycomb-group complex [46,47] and a known transcriptional repressor. SMAD2/3 and RUNX2 have been reported to act as both activators and repressors [50,66]. Accordingly, many of the genes associated with this RUNX2/BMI-1/SMAD2/3 complex in BG01V cells (for example *SMA*, *IFNG*, *GATA4 *and *6*, *MUC4*, *FOS*, *SNAIL*, *P21*; data not shown) are not expressed in undifferentiated ES cells [38,54], suggesting that this RUNX2/BMI-1/SMAD2/3 complex plays a transcriptional repressor role in undifferentiated BG01V cells.

## Conclusion

In summary, our simplified basic ChIP and novel Sequential ChIP protocols were successfully tested with a variety of antibodies and generated a number of novel observations for future studies. The basic ChIP assay combines simplicity (avoidance of several commonly used steps), speed (whole assay performed in 2 hours) and sensitivity (using 20,000 cells). More importantly, promoter co-occupancy data obtained with our novel Sequential ChIP protocol indicates the existence of two TF complexes in human ES cells: SOX2/OCT3/4/NANOG/c-MYC and RUNX2/BMI-1/SMAD2/3. This novel Sequential ChIP protocol provides a significant improvement from previously described Sequential ChIP assays by combining two methods of physical separation of the targeted DNA (centrifugation and magnetism) and avoiding antibody-antigen interaction disruption during the assay. Both protocols might be useful for high-throughput ChIP-based assays, which are expensive, labor intensive, require complex statistical analysis, and might greatly benefit from faster and straightforward ChIP assay and analysis methodologies.

## Methods

### Cells and cell culture

Mouse ES cell line D3 was purchased from American Type Culture Collection (ATCC) and cultured according to the manufacturer's instructions. Undifferentiated human ES cell line BG01V (Novocell) were cultured according to the manufacturer's instructions, without feeder cells [38]. Endoderm differentiation of D3 cells was performed as described previously [67].

### Immunocytochemistry

Primary antibodies were used at 10 μg/ml. BG01V cells were cultured in 24-well plates, fixed with 4% paraformaldehyde/PBS at room temperature (RT) for 20 min, blocked and permeabilized with 0.1% Triton X-100, 1% BSA, and 10% normal donkey serum in PBS, at RT, for 45 min. After blocking, cells were incubated with diluted primary antibody overnight at 4°C, followed by 5 μg/ml secondary antibody (anti-IgG-NL557, R&D Systems) at RT, in the dark, for 1 hour. Cells were washed with PBS/0.1% BSA between each step. No staining was obtained with secondary antibody only, used as a negative control (data not shown).

### Reagents and antibodies

Anti-SOX2, NANOG, OCT3/4, c-MYC, PDX1, OLIG2, RUNX2, SMAD2/3, KLF4, ERK1/2 and BMI-1 antibodies, streptavidin magnetic beads (R&D Systems); antibody specificity was determined previously with appropriate controls by Western blotting and immunocytochemistry [ref. [13] and data not shown]; Odyssey western blot blocker buffer (LI-COR Biosciences); IDRdye 800 anti-goat IgG antibody (Rockland Immunochemicals); phorbol-12-myristate-13-acetate (PMA), Chelex-100, lithium chloride, sodium deoxycholate, leupeptin, phenyl-methanessulfonyl-floride (PMSF), 37% formaldehyde, NP-40, glycine, dimethyl-sulfoxide (DMSO), DAPI, aprotinin (Sigma); AmpliTaq^® ^Gold Polymerase PCR kit, dNTP mix (Applied Biosystems); EXPRESS SYBR^® ^GreenER™ qPCR kit (Invitrogen); QIAQuick^® ^DNA purification kit (Qiagen); 96-well PCR plates, 96-well plate foil (Eppendorf), protein A-agarose beads (Thermo Scientific), and protein G-agarose beads (Pierce).

### Western blotting and Immunoprecipitation

Cells were lysed with 2× lysis buffer (2% NP-40, 100 mM Tris-HCl, 300 mM NaCl, 4 mM EDTA, 2 mM sodium vanadate, 10 μg/ml leupeptin, 2 mM PMSF, 10 μg/ml aprotinin) and equal number of cell equivalents were separated by 10% SDS-PAGE. After Western transfer, nitrocellulose membranes were blocked with Odyssey blocker/PBS (1:1) for 1 hour, incubated with primary antibodies for 30 min in Odyssey blocker/PBS/0.2% Tween, washed with PBS/0.2% Tween 3× for 5 min, incubated for 30 min with secondary fluorescent antibody (IDRdye 800 donkey anti-goat IgG) in Odyssey blocker/PBS/0.2%Tween/0.02%SDS, and washed with PBS/0.2%Tween 3× for 5 min. Fluorescence was detected and images captured with an Odyssey infrared imager (LI-COR Biosciences). For immunoprecipitation (IP) lysates were incubated with 5 μg of biotinylated anti-SOX2 antibody for 4 hrs at 4°C, for each round of IP. Samples were incubated with 20 μL of streptavidin magnetic beads (R&D Systems) for 30 min at 4°C, beads were collected with a magnet, and washed with wash buffer (20 mM HEPES pH 7.6, 50 mM NaCl, 2.5 mM MgCl_2_, 0.1 mM EDTA, and 0.05% Triton X-100).

### Basic ChIP Protocol

The basic ChIP assay (diagram shown in Fig. 1) is a combination of sensitive and fast ChIP assays described previously [11,12] with some modifications. Cells were fixed with 1% formaldehyde for 15 min (RT), quenched with 125 mM glycine for 5 min (RT), centrifuged and resuspended in Lysis Buffer (1% SDS, 10 mM EDTA, 50 mM Tris, pH 8) containing protease inhibitors (10 μg/mL leupeptin, 10 μg/mL aprotinin, and 1 mM PMSF) and incubated on ice (10 min). Initial samples consisted of about 1 × 10^6 ^cells in 0.5 mL of Lysis Buffer. Next samples were sonicated (Heat Systems-Ultrasonic device) to shear chromatin to an average length of about 1 kb and transferred to 1.5 mL tubes, microcentrifuged for 10 min (max speed). Supernatants were collected in 1.5 mL tubes containing 1 mL of the Dilution Buffer (0.01% SDS, 1.1% Triton, 1.2 mM EDTA, 167 mM NaCl, 17 mM Tris, pH 8). Five μg of biotinylated primary antibodies were added, samples were incubated (15 min) in an ultrasonic bath (Branson), followed by addition of 50 μL of streptavidin magnetic beads, incubated (30 min, 4°C) on a rotator (Labnet Int.). Beads were collected with a magnet (R&D Systems), washed 4× with 1 mL of each of four Wash Buffers (Wash Buffer 1: 0.1% SDS, 1% Triton, 2 mM EDTA, 150 mM NaCl, 20 mM Tris, pH 8; Wash Buffer 2: 0.1% SDS, 1% Triton, 2 mM EDTA, 500 mM NaCl, 20 mM Tris, pH 8; Wash Buffer 3: 0.25 M LiCl, 1% NP-40, 1% deoxycholate, 1 mM EDTA, 10 mM Tris, pH 8; Wash Buffer 4: 10 mM Tris, pH 8, 1 mM EDTA). After the last wash, 100 μL of a 10% Chelex-100 (Sigma)/PBS resin solution was added to the beads, samples were boiled (10 min) in a heat block, microcentrifuged (1 min, max speed), supernatants transferred to a 1.5 mL tube, followed by the addition of 120 μL of MilliQ water back to the beads, microcentrifuged again (1 min, max speed), and the new supernatant pooled with the previous one. DNA samples were then cleaned up with QIAQuick kit^®^, resuspended in 50 μL of Wash Buffer 4 and 1 μL (containing about 20,000 cell equivalents) was used per PCR reaction. Experiments were performed 3×, with independent samples.

### Sequential ChIP

The Sequential ChIP protocol (diagram shown in Fig. 5) was performed as follows. Samples were treated as above until addition of the first antibody. Five μg of the first antibody was added, this time a non-biotinylated antibody, incubated for 2 hrs to overnight at 4°C, followed by 50 μL of protein G-agarose beads for 30 min at 4°C on a rotator (Labnet Int.). Samples were then washed as described above, using a microcentrifuge instead of a magnet to pull-down the agarose beads. Subsequently the agarose beads were resuspended in 1 mL of Dilution buffer. Five μg of the second antibody was added, this time a biotinylated antibody, incubated for 2 hrs to overnight at 4°C, followed by incubation with magnetic streptavidin beads. Beads were washed as described above, using a magnet, and the final DNA samples were obtained as described above (basic ChIP protocol). Experiments were performed 3×, with independent samples.

### PCR and Densitometric Image Analysis

DNA samples, from ChIP assays or input DNA (0.1% of the sample used for the ChIP assay), primers (1 μM, IDT) and DMSO (5%) were added to the additional PCR reagents (AmpliTaq^® ^Gold Polymerase PCR kit and dNTP mix) in a 50 μL reaction volume and subjected to the following cycle: 95°C/9 min, 43× (95°C/1 min, 60°C/1 min), and 60°C/10 min, using 96-well plates and an Eppendorf Mastercycler^®^. PCR products were subjected to a 1.5% agarose gel electrophoresis, visualized with Ethidium bromide staining and photographed. Images were saved as TIFF files for analysis with ImageJ. All primers have been validated previously (Table 1). Signal intensities from PCR data obtained from ChIP assays or from whole cell lysates (Input DNA) were quantified from the TIFF images with ImageJ software (National Institutes of Health; ), as previously described [68] with some modifications. Initially images were transformed to 16-bit-type images. The threshold function was set to black and white type of image to eliminate background interference. Threshold limits were set to compensate possible saturation interference. The rectangle tool was used to draw the area for measurement around the PCR bands and the same rectangle tool was moved to each band in the blot. Area, area fraction and mean gray values were taken. Mean gray values were used as a measure of relative number of pixels. Fold enrichment = ChIP/Input DNA. For each TF three independent ChIP experiments were performed. Average and standard deviations (SD) were calculated based on the signal intensities from each experiment.

### Real-time PCR (rtPCR)

DNA samples, from ChIP assays or input DNA (0.1% of the sample used for the ChIP assay), and primers (200 nM, IDT) were added to the additional rtPCR reagents (EXPRESS SYBR^® ^GreenER™ qPCR kit) in a 20 μL reaction volume and subjected to the following cycle: 50°C/2 min, 95°C/2 min, 40× (95°C/15 sec, 60°C/1 min), using ABI 7900 HT or Cepheid Smart rtPCR instruments. No template (primers only) reactions were used for normalization. Each sample was amplified in triplicate. Three independent experiments were performed. Average and SD were calculated based on relative copy numbers from each experiment.

## Authors' contributions

RM.: conception and design, data collection, analysis and interpretation, manuscript writing; KP, BH, KC, JJ, S-kG, AE: data collection; ER, JH: conception and design, data analysis and interpretation; H-TN: financial support, administrative support, conception and design, data analysis and interpretation. All authors read and approved the final manuscript.

## References

[B1] Levine M, Tjian R (2003). Transcription regulation and animal diversity. Nature.

[B2] Meng X, Brodsky MH, Wolfe SA (2005). A bacterial one-hybrid system for determining the DNA-binding specificity of transcription factors. Nat Biotechnol.

[B3] Mukherjee S, Berger MF, Jona G, Wang XS, Muzzey D, Snyder M, Young RA, Bulyk ML (2004). Rapid analysis of the DNA-binding specificities of transcription factors with DNA microarrays. Nat Genet.

[B4] Walhout AJ (2006). Unraveling transcription regulatory networks by protein-DNA and protein-protein interaction mapping. Genome Res.

[B5] Solomon MJ, Larsen PL, Varshavsky A (1988). Mapping protein-DNA interactions in vivo with formaldehyde: evidence that histone H4 is retained on a highly transcribed gene. Cell.

[B6] Ren B, Robert F, Wyrick JJ, Aparicio O, Jennings EG, Simon I, Zeitlinger J, Schreiber J, Hannett N, Kanin E, Volkert TL, Wilson CJ, Bell SP, Young RA (2000). Genome-wide location and function of DNA binding proteins. Science.

[B7] Kim TH, Ren B (2006). Genome-wide analysis of protein-DNA interactions. Annu Rev Genomics Hum Genet.

[B8] Robertson G, Hirst M, Bainbridge M, Bilenky M, Zhao Y, Zeng T, Euskirchen G, Bernier B, Varhol R, Delaney A, Thiessen N, Griffith OL, He A, Marra M, Snyder M, Jones S (2007). Genome-wide profiles of STAT1 DNA association using chromatin immunoprecipitation and massively parallel sequencing. Nat Methods.

[B9] Collas P, Dahl JA (2008). Chop it, ChIP it, check it: the current status of chromatin immunoprecipitation. Front Biosci.

[B10] Sharov AA, Masui S, Sharova LV, Piao Y, Aiba K, Matoba R, Xin L, Niwa H, Ko MS (2008). Identification of Pou5f1, Sox2, and Nanog downstream target genes with statistical confidence by applying a novel algorithm to time course microarray and genome-wide chromatin immunoprecipitation data. BMC Genomics.

[B11] Nelson JD, Denisenko O, Bomsztyk K (2006). Protocol for the fast chromatin immunoprecipitation (ChIP) method. Nat Protoc.

[B12] Dahl JA, Collas P (2008). A rapid micro chromatin immunoprecipitation assay (microChIP). Nat Protoc.

[B13] Cai J, Olson JM, Rao MS, Stanley M, Taylor E, Ni HT (2005). Development of antibodies to human embryonic stem cell antigens. BMC Dev Biol.

[B14] Masui S, Nakatake Y, Toyooka Y, Shimosato D, Yagi R, Takahashi K, Okochi H, Okuda A, Matoba R, Sharov AA, Ko MS, Niwa H (2007). Pluripotency governed by Sox2 via regulation of Oct3/4 expression in mouse embryonic stem cells. Nat Cell Biol.

[B15] Su L, David M (2000). Distinct mechanisms of STAT phosphorylation via the interferon-alpha/beta receptor. Selective inhibition of STAT3 and STAT5 by piceatannol. J Biol Chem.

[B16] Nelson EA, Walker SR, Alvarez JV, Frank DA (2004). Isolation of unique STAT5 targets by chromatin immunoprecipitation-based gene identification. J Biol Chem.

[B17] Barlev NA, Liu L, Chehab NH, Mansfield K, Harris KG, Halazonetis TD, Berger SL (2001). Acetylation of p53 activates transcription through recruitment of coactivators/histone acetyltransferases. Mol Cell.

[B18] Kirmizis A, Bartley SM, Farnham PJ (2003). Identification of the polycomb group protein SU(Z)12 as a potential molecular target for human cancer therapy. Mol Cancer Ther.

[B19] Jonckheere N, Vincent A, Perrais M, Ducourouble MP, Male AK, Aubert JP, Pigny P, Carraway KL, Freund JN, Renes IB, Van Seuningen I (2007). The human mucin MUC4 is transcriptionally regulated by caudal-related homeobox, hepatocyte nuclear factors, forkhead box A, and GATA endodermal transcription factors in epithelial cancer cells. J Biol Chem.

[B20] Nandiwada SL, Li W, Zhang R, Mueller DL (2006). p300/Cyclic AMP-responsive element binding-binding protein mediates transcriptional coactivation by the CD28 T cell costimulatory receptor. J Immunol.

[B21] Cao R, Tsukada Y, Zhang Y (2005). Role of Bmi-1 and Ring1A in H2A ubiquitylation and Hox gene silencing. Mol Cell.

[B22] Hu MC, Mo R, Bhella S, Wilson CW, Chuang PT, Hui CC, Rosenblum ND (2006). GLI3-dependent transcriptional repression of Gli1, Gli2 and kidney patterning genes disrupts renal morphogenesis. Development.

[B23] Regl G, Kasper M, Schnidar H, Eichberger T, Neill GW, Philpott MP, Esterbauer H, Hauser-Kronberger C, Frischauf AM, Aberger F (2004). Activation of the BCL2 promoter in response to Hedgehog/GLI signal transduction is predominantly mediated by GLI2. Cancer Res.

[B24] Peinado H, Olmeda D, Cano A (2007). Snail, Zeb and bHLH factors in tumour progression: an alliance against the epithelial phenotype?. Nat Rev Cancer.

[B25] Yu P, Kodadek T (2007). Dynamics of the hypoxia-inducible factor-1-vascular endothelial growth factor promoter complex. J Biol Chem.

[B26] Peiro S, Escriva M, Puig I, Barbera MJ, Dave N, Herranz N, Larriba MJ, Takkunen M, Franci C, Munoz A, Virtanen I, Baulida J, Garcia de Herreros A (2006). Snail1 transcriptional repressor binds to its own promoter and controls its expression. Nucleic Acids Res.

[B27] Escriva M, Peiro S, Herranz N, Villagrasa P, Dave N, Montserrat-Sentis B, Murray SA, Franci C, Gridley T, Virtanen I, Garcia de Herreros A (2008). Repression of PTEN phosphatase by Snail1 transcriptional factor during gamma radiation-induced apoptosis. Mol Cell Biol.

[B28] Tang Y, Urs S, Liaw L (2008). Hairy-related transcription factors inhibit Notch-induced smooth muscle alpha-actin expression by interfering with Notch intracellular domain/CBF-1 complex interaction with the CBF-1-binding site. Circ Res.

[B29] Furumatsu T, Tsuda M, Taniguchi N, Tajima Y, Asahara H (2005). Smad3 induces chondrogenesis through the activation of SOX9 via CREB-binding protein/p300 recruitment. J Biol Chem.

[B30] Caslini C, Capo-chichi CD, Roland IH, Nicolas E, Yeung AT, Xu XX (2006). Histone modifications silence the GATA transcription factor genes in ovarian cancer. Oncogene.

[B31] Eliseev RA, Dong YF, Sampson E, Zuscik MJ, Schwarz EM, O'Keefe RJ, Rosier RN, Drissi MH (2008). Runx2-mediated activation of the Bax gene increases osteosarcoma cell sensitivity to apoptosis. Oncogene.

[B32] Liu G, Ding W, Neiman J, Mulder KM (2006). Requirement of Smad3 and CREB-1 in mediating transforming growth factor-beta (TGF beta) induction of TGF beta 3 secretion. J Biol Chem.

[B33] Saifudeen Z, Dipp S, Fan H, El-Dahr SS (2005). Combinatorial control of the bradykinin B2 receptor promoter by p53, CREB, KLF-4, and CBP: implications for terminal nephron differentiation. Am J Physiol Renal Physiol.

[B34] Chen C, Rowell EA, Thomas RM, Hancock WW, Wells AD (2006). Transcriptional regulation by Foxp3 is associated with direct promoter occupancy and modulation of histone acetylation. J Biol Chem.

[B35] Wang ZX, Teh CH, Kueh JL, Lufkin T, Robson P, Stanton LW (2007). Oct4 and Sox2 directly regulate expression of another pluripotency transcription factor, Zfp206, in embryonic stem cells. J Biol Chem.

[B36] Chakravarthy H, Boer B, Desler M, Mallanna SK, McKeithan TW, Rizzino A (2008). Identification of DPPA4 and other genes as putative Sox2:Oct-3/4 target genes using a combination of in silico analysis and transcription-based assays. J Cell Physiol.

[B37] Patterson ES, Addis RC, Shamblott MJ, Gearhart JD (2008). SOX17 directly activates Zfp202 transcription during in vitro endoderm differentiation. Physiol Genomics.

[B38] Zeng X, Miura T, Luo Y, Bhattacharya B, Condie B, Chen J, Ginis I, Lyons I, Mejido J, Puri RK, Rao MS, Freed WJ (2004). Properties of pluripotent human embryonic stem cells BG01 and BG02. Stem Cells.

[B39] Boyer LA, Lee TI, Cole MF, Johnstone SE, Levine SS, Zucker JP, Guenther MG, Kumar RM, Murray HL, Jenner RG, Gifford DK, Melton DA, Jaenisch R, Young RA (2005). Core transcriptional regulatory circuitry in human embryonic stem cells. Cell.

[B40] Kanai-Azuma M, Kanai Y, Gad JM, Tajima Y, Taya C, Kurohmaru M, Sanai Y, Yonekawa H, Yazaki K, Tam PP, Hayashi Y (2002). Depletion of definitive gut endoderm in Sox17-null mutant mice. Development.

[B41] Niimi T, Hayashi Y, Futaki S, Sekiguchi K (2004). SOX7 and SOX17 regulate the parietal endoderm-specific enhancer activity of mouse laminin alpha1 gene. J Biol Chem.

[B42] Komori T (2008). Regulation of bone development and maintenance by Runx2. Front Biosci.

[B43] Zelzer E, Glotzer DJ, Hartmann C, Thomas D, Fukai N, Soker S, Olsen BR (2001). Tissue specific regulation of VEGF expression during bone development requires Cbfa1/Runx2. Mech Dev.

[B44] Liu Y, Sinha S, McDonald OG, Shang Y, Hoofnagle MH, Owens GK (2005). Kruppel-like factor 4 abrogates myocardin-induced activation of smooth muscle gene expression. J Biol Chem.

[B45] Jiang J, Chan YS, Loh YH, Cai J, Tong GQ, Lim CA, Robson P, Zhong S, Ng HH (2008). A core Klf circuitry regulates self-renewal of embryonic stem cells. Nat Cell Biol.

[B46] Jacobs JJ, Kieboom K, Marino S, DePinho RA, van Lohuizen M (1999). The oncogene and Polycomb-group gene bmi-1 regulates cell proliferation and senescence through the ink4a locus. Nature.

[B47] Molofsky AV, He S, Bydon M, Morrison SJ, Pardal R (2005). Bmi-1 promotes neural stem cell self-renewal and neural development but not mouse growth and survival by repressing the p16Ink4a and p19Arf senescence pathways. Genes Dev.

[B48] Roberts AB, Tian F, Byfield SD, Stuelten C, Ooshima A, Saika S, Flanders KC (2006). Smad3 is key to TGF-beta-mediated epithelial-to-mesenchymal transition, fibrosis, tumor suppression and metastasis. Cytokine Growth Factor Rev.

[B49] Garcia-Campmany L, Marti E (2007). The TGFbeta intracellular effector Smad3 regulates neuronal differentiation and cell fate specification in the developing spinal cord. Development.

[B50] Brown KA, Pietenpol JA, Moses HL (2007). A tale of two proteins: differential roles and regulation of Smad2 and Smad3 in TGF-beta signaling. J Cell Biochem.

[B51] Ligon KL, Fancy SP, Franklin RJ, Rowitch DH (2006). Olig gene function in CNS development and disease. Glia.

[B52] Fellous TG, Guppy NJ, Brittan M, Alison MR (2007). Cellular pathways to beta-cell replacement. Diabetes/Metabolism Res and Rev.

[B53] Zeller KI, Jegga AG, Aronow BJ, O'Donnell KA, Dang CV (2003). An integrated database of genes responsive to the Myc oncogenic transcription factor: identification of direct genomic targets. Genome Biol.

[B54] Kidder BL, Yang J, Palmer S (2008). Stat3 and c-Myc genome-wide promoter occupancy in embryonic stem cells. PLoS ONE.

[B55] Kim J, Chu J, Shen X, Wang J, Orkin SH (2008). An extended transcriptional network for pluripotency of embryonic stem cells. Cell.

[B56] van Werven FJ, Marc Timmers HTh (2006). The use of biotin tagging in Sccharomyces cerevisiae improves the sensitivity of chromatin immunoprecipitation. Nucleic Acids Res.

[B57] Scully KM, Jacobson EM, Jepsen K, Lunyak V, Viadiu H, Carriere C, Rose DW, Hooshmand F, Aggarwal AK, Rosenfeld MG (2000). Allosteric effects of Pit-1 DNA sites on long-term repression in cell type specification. Science.

[B58] Metivier R, Penot G, Hubner MR, Reid G, Brand H, Kos M, Gannon F (2003). Estrogen receptor-alpha directs ordered, cyclical, and combinatorial recruitment of cofactors on a natural target promoter. Cell.

[B59] Geisberg JV, Struhl K (2004). Quantitative sequential chromatin immunoprecipitation, a method for analyzing co-occupancy of proteins at genomic regions in vivo. Nucleic Acids Res.

[B60] Geisberg JV, Struhl K (2004). Cellular stress alters the transcriptional properties of promoter-bound Mot1-TBP complexes. Mol Cell.

[B61] Patel JH, Du Y, Ard PG, Phillips C, Carella B, Chen CJ, Rakowski C, Chatterjee C, Lieberman PM, Lane WS, Blobel GA, McMahon SB (2004). The c-MYC oncoprotein is a substrate of the acetyltransferases hGCN5/PCAF and TIP60. Mol Cell Biol.

[B62] Fernandez PC, Frank SR, Wang L, Schroeder M, Liu S, Greene J, Cocito A, Amati B (2003). Genomic targets of the human c-Myc protein. Genes Dev.

[B63] Amati B, Frank SR, Donjerkovic D, Taubert S (2001). Function of the c-Myc oncoprotein in chromatin remodeling and transcription. Biochim Biophys Acta.

[B64] Selvamurugan N, Kwok S, Alliston T, Reiss M, Partridge NC (2004). Transforming growth factor-beta 1 regulation of collagenase-3 expression in osteoblastic cells by cross-talk between the Smad and MAPK signaling pathways and their components, Smad2 and Runx2. J Biol Chem.

[B65] Hanai J, Chen LF, Kanno T, Ohtani-Fujita N, Kim WY, Guo WH, Imamura T, Ishidou Y, Fukuchi M, Shi MJ, Stavnezer J, Kawabata M, Miyazono K, Ito Y (1999). Interaction and functional cooperation of PEBP2/CBF with Smads. Synergistic induction of the immunoglobulin germline Calpha promoter. J Biol Chem.

[B66] Jensen ED, Nair AK, Westendorf JJ (2007). Histone deacetylase co-repressor complex control of Runx2 and bone formation. Crit Rev Eukaryot Gene Expr.

[B67] D'Amour KA, Bang AG, Eliazer S, Kelly OG, Agulnick AD, Smart NG, Moorman MA, Kroon E, Carpenter MK, Baetge EE (2006). Production of pancreatic hormone-expressing endocrine cells from human embryonic stem cells. Nat Biotechnol.

[B68] Chapman MD, Keir G, Petzold A, Thompson EJ (2006). Measurement of high affinity antibodies on antigen-immunoblots. J Immunol Methods.

